# Effect of proton pump inhibitors in infants with esophageal atresia on the gut microbiome: a pilot cohort

**DOI:** 10.1186/s13099-022-00518-9

**Published:** 2022-12-16

**Authors:** Nele Brusselaers, Marcela Pereira, Johan Alm, Lars Engstrand, Helene Engstrand Lilja

**Affiliations:** 1 Centre for Translational Microbiome Research, Department of Microbiology, Tumor and Cell Biology, Karolinska Institutet, Solnavägen 9, 17165 Stockholm, Sweden; 2 Global Health Institute, University of Antwerp, B-2610 Antwerp, Belgium; 3 Department of Head and Skin, Ghent University, B-9000 Ghent, Belgium; 4 Department of Clinical Science, Karolinska Institutet, 17165 Stockholm, Sweden; 5 Sachs’ Children and Youth Hospital, Södersjukhuset, 11883 Stockholm, Sweden; 6 Department of Women’s and Children’s Health, Karolinska Institutet, 17165 Stockholm, Sweden

**Keywords:** Esophageal atresia, Microbiome, PPI, Proton pump inhibitor, Gastric acid, Infants

## Abstract

**Background:**

The effects of proton-pump inhibitors (PPIs) on the infant microbiome remain unclear. Swedish pilot cohort study to assess the longitudinal effect of long-term PPI on the infant gut microbiome, including ten newborn infants operated for esophageal atresia exposed to PPIs (mean 57 weeks), compared to healthy one-year-old controls. All children were born vaginally and were otherwise healthy. Within- and between sample diversity of the fecal microbiome was assessed using untargeted whole genome Shotgun metagenomics which sequences all the DNA in the sample and can capture genes rather than a taxonomic fingerprint.

**Results:**

A longer duration of PPI-use was associated with considerable changes in evenness and high variation on diversity within samples compared to a shorter duration of use. The limited difference between baseline samples and controls suggests that this shift was most likely due to the drug exposure and not the underlying alterations on the microbiome. We found no associations with the number of antibiotic treatment episodes among the PPI-users.

**Conclusion:**

Prolonged PPI-use may alter the early infant gut microbiome composition, especially those with the most prolonged duration of use.

**Supplementary Information:**

The online version contains supplementary material available at 10.1186/s13099-022-00518-9.

## Background

Proton pump inhibitors (PPIs) are gastric acid-suppressive medication, frequently (over-) prescribed and used in all age-groups [1–5], also during the first years of life [6–11]. Although there is no clear consensus on the indications or duration of use in pediatrics [12], PPIs are among the most common off-label used medications in infants and young children [10, 13, 14]. The broad range of pediatric and especially neonatal indications include (presumed) gastro-esophageal or laryngopharyngeal reflux, prevention and treatment of stress ulcers, eosinophilic esophagitis and infantile colic [12, 15, 16]. European and American guidelines recommend treatment for at least one year after surgical repair of esophageal atresia (EA), a congenital malformation characterized by a lost continuity between the upper and lower esophagus, to reduce the risk of esophageal strictures [17]. Despite the relatively stable prevalence of these disorders over the last decades, PPIs have been increasingly used in out- and inpatient settings [2, 4, 18–20], including children [9, 21, 22].

Yet, pediatric PPIs’ efficacy and safety have been repeatedly questioned, including in EA [11, 23–26]. PPI use in children may be associated with an increased risk of adverse events in the gastrointestinal tract (including eosinophilic esophagitis*, Clostridioides difficile* infections, necrotizing enterocolitis, diarrhea, constipation, and gastroenteritis), sepsis, pneumonia, asthma, other allergic diseases and hypomagnesemia [11, 27–32]. Adult studies suggest an even broader list of potential long-term consequences of maintenance PPI use, including cancer, osteoporosis, acute and chronic kidney disease and even poorer overall survival [33–36]. Although not recommended in pregnancy, maternal PPI use has also been associated with an increased risk of congenital malformations, preterm birth, being born small for gestational age, preeclampsia, gestational diabetes and even childhood asthma [37–39]. Altogether, the large and unwarranted scale of PPI use may result in a high burden on population level [33, 40]. Although these potential long-term consequences remain challenging to study (requiring large numbers, proper adjustment for confounding and long follow-up), and the effect sizes may seem relatively limited, one emerging hypothesis which may explain part of these seemingly diverse and incoherent associations is the effect of PPIs on the microbiome.

It is presumed that the first years of life are crucial to establish a healthy microbiome and consequently reduce the risk of several diseases [41–43]. From adult studies, we know that PPIs may have the most significant impact on the fecal microbiome composition on a population level, even larger than antibiotics which are rarely used for such a prolonged duration; and that PPIs may affect the microbiome throughout the gastrointestinal tract [44–47]. We also know that antibiotic exposure during early life affects the microbiome composition [48–53], yet only a few studies looked into the effect of PPIs on the early gut microbiome [29, 54, 55].

Therefore, the present pilot study aimed to assess the effect of long-term PPI on the intestinal microbiome in infants operated for EA—accounting for duration of use.

## Results

Of the 20 infants with EA Gross type C participating in the one-year follow up, one was excluded because of C-section delivery, three as they were treated with antibiotics within 3 months before sampling and two were excluded because fewer than the three samples required for the study were collected. Four families were excluded as they never collected fecal samples from their child at the first timepoint.

**Fig. 1:**
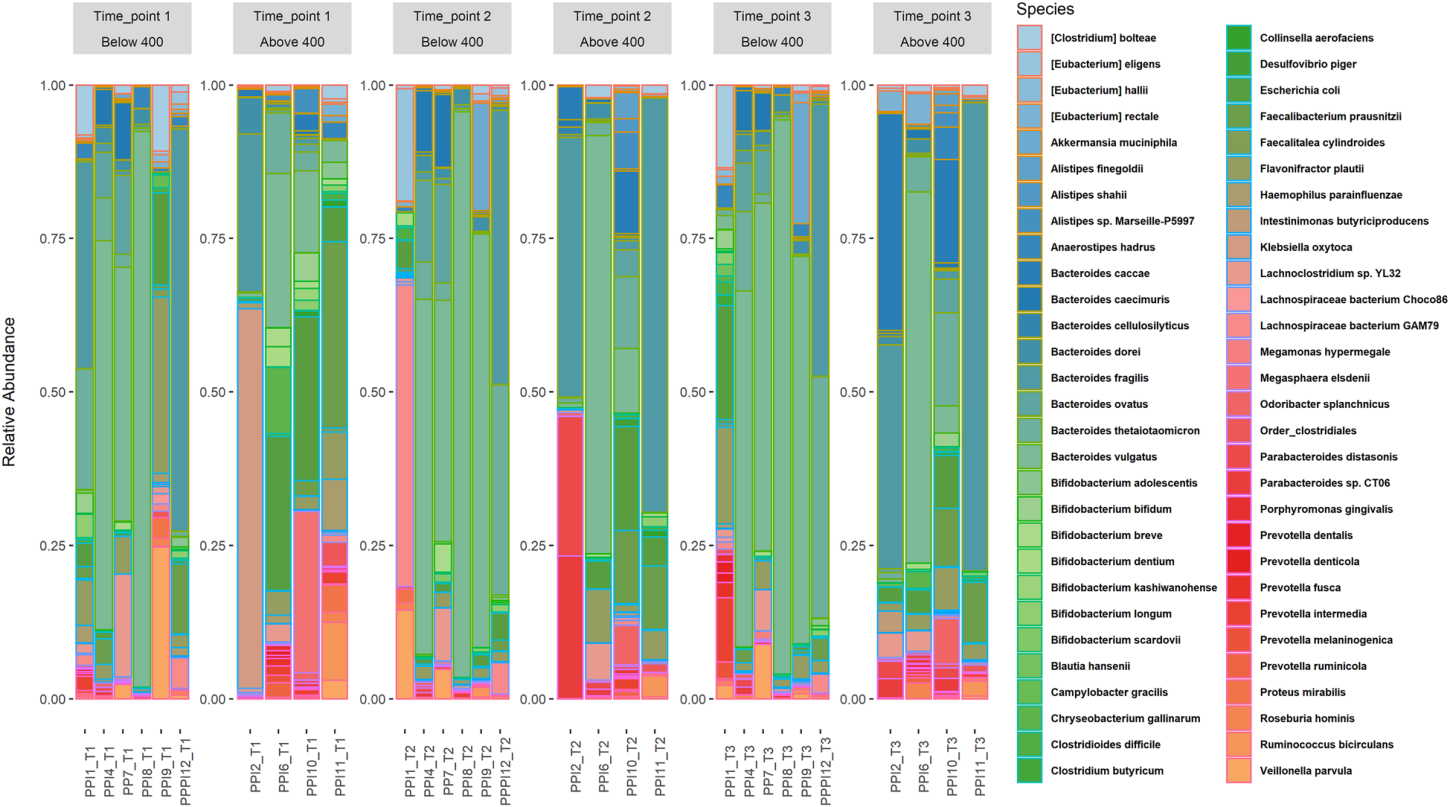
Stacked barplot showing Control versus Proton Pump Inhibitor (PPI) groups at species level at the different timepoints. Timepoint 1 (T1) is the baseline condition for PPI treatment; Timepoint 2 (T2) is the end of PPI treatment, and Timepoint 3 (T3) is 1 month after PPI treatment. Data are divided according to PPI duration, above or below 400 days

Of the remaining ten children with EA, five were male, and all were delivered vaginally. The gestational age at the time of delivery ranged from 35 to 41 weeks. Surgical repair occurred at 1–4 days of age, and PPIs were initiated on days 1–7 after surgery. The duration of PPI use ranged from 347 to 475 days (mean 401 days or 57 weeks), with six children receiving PPIs less than 400 days. Figure 1 and Additional file 1: Fig, S1 show barplots of the different timepoints (baseline and follow-up) comparing cases and controls.

All included infants with EA received antibiotics during at least one period (7–13 days), starting on the day of birth or the day after, so 0–3 days before the surgery. Five infants received two or more antibiotic treatments, with maximally four antibiotic treatment episodes during the first year of life.

The 12 children from the ALADDIN cohort included four boys and eight girls who did not receive antibiotics or PPIs.

### Alpha-diversity in all infants on PPI use

**Fig. 2:**
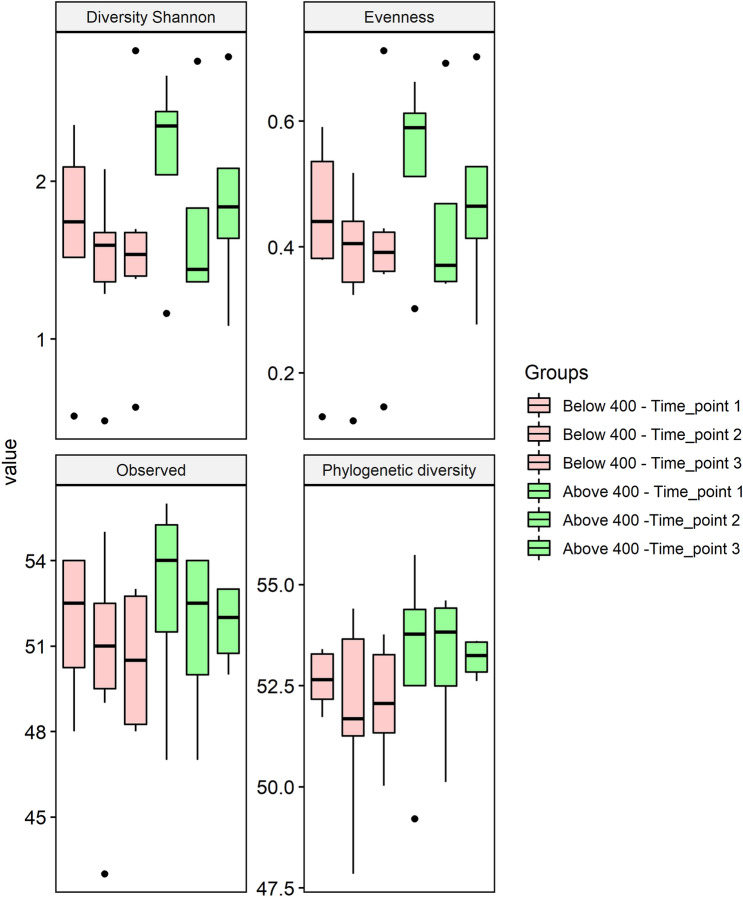
Alpha diversity of baseline data in the total group of proton pump inhibitor (PPI) users by treatment duration (below or above 400 days). Top left: Observed metrics; Top Right: Shannon Diversity; Bottom left: Pielou Evenness; Bottom right: Phylogenetic Diversity

A longitudinal analysis of each timepoint per group (below or above 400 days) did not show statistical difference between time points. Although visually it is possible to observe a higher variability along time points, for Shannon diversity and evenness analysis for the group above 400 days. In this group, a substantial drop was observed on timepoint two, and it did not recover by the last timepoint. These differences were not statistically significant, most likely due to the small cohort and high variability (outliers) (Fig. 2). A more stable profile seemed to be reached when the PPI treatment was not prolonged for more than 400 days; it seemed to stabilize with only a few species disappearing over time when observed longitudinally. While for the phylogenetic diversity, no differences were observed, and the values were constant in both groups along the timepoints. In opposition, compared to the control group, a lower phylogenetic diversity was found among PPI users at baseline condition (Additional file 2: Fig. S2), and a decrease in Shannon and evenness diversity was observed at the end of PPI for both groups at the latest timepoint (Additional file 3: Fig. S3). The statistical comparisons (Additional file 10: Table S1b) of control versus a single time point, ANOVA followed by Tukey post-hoc was used (since the data presented normality when tested by Shapiro’s test), for longitudinal analysis was applied a linear mixed model.

### Beta-diversity

**Fig. 3:**
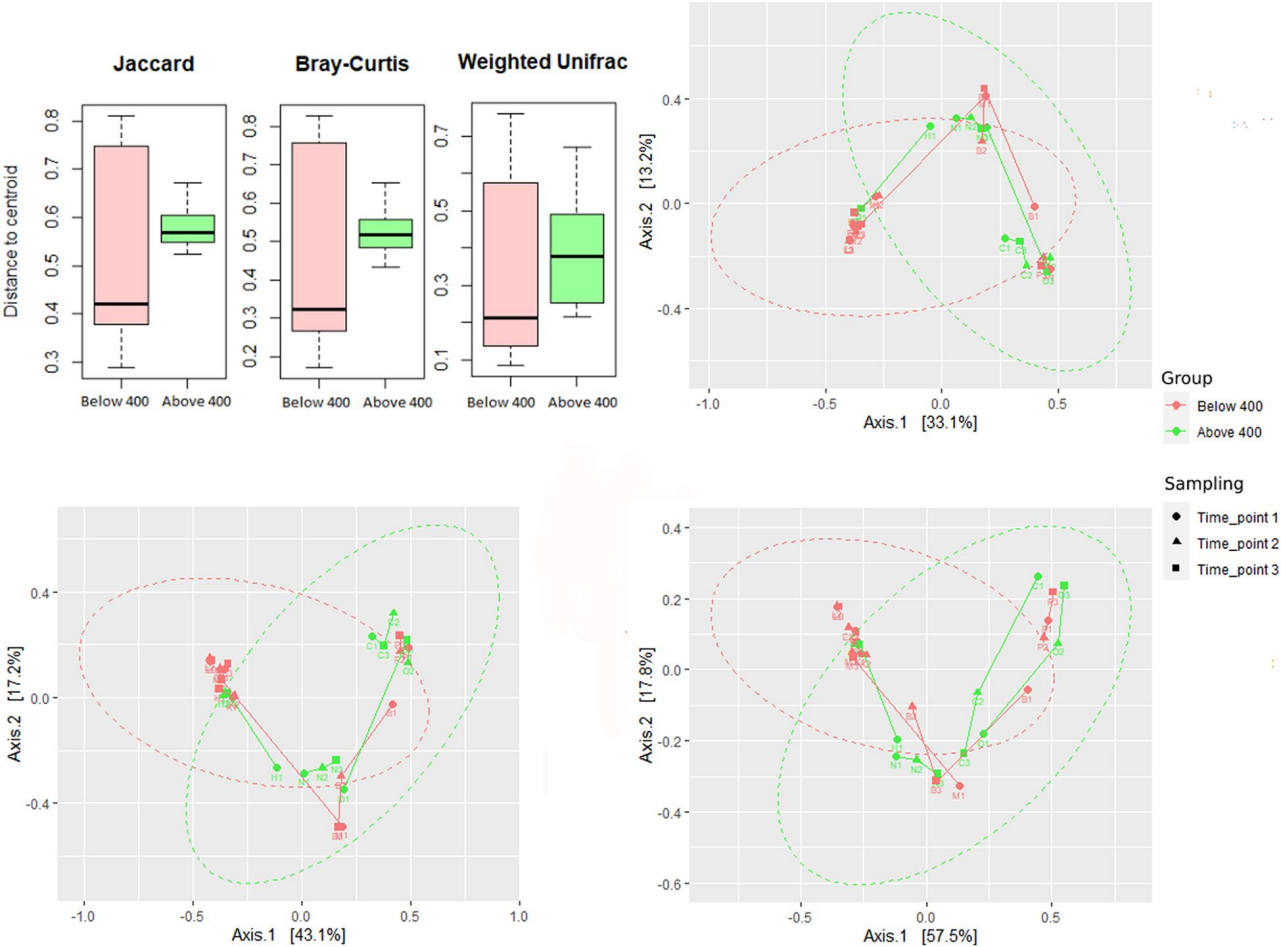
Beta diversity of the total group exposed to proton pump inhibitors (PPI) (all timepoints), by treatment duration (below or above 400 days) and by timepoint of sampling. Top left: dispersion of samples from centroid point for specific beta diversity metrics. Top right: PCoA plot of bray Curtis matrix; Bottom left: PCoA plot of Jaccard; Bottom right: PCoA plot of weighted unifrac

Beta-diversity analyses showed a larger variation in the group exposed to PPIs for less than 400 days than in the group with a more prolonged exposure (Fig. 3a), suggesting that some infants are more affected than others in the low PPI group or that the starting diversity was more variable in this group than in the longer PPI duration group. The PCoA plots (Fig. 3b–d) did suggest clustering when assessing all PPI using infants (low and high duration), yet this did not reach statistical significance. By looking at the different timepoints and the trajectory of the samples (lanes connecting the same sample in different timepoints), the data suggest that for most of the subjects receiving PPI for a longer duration, there was a higher variability in diversity. While for the group that received PPI for a shorter period, only two samples presented such characteristics.

**Fig. 4:**
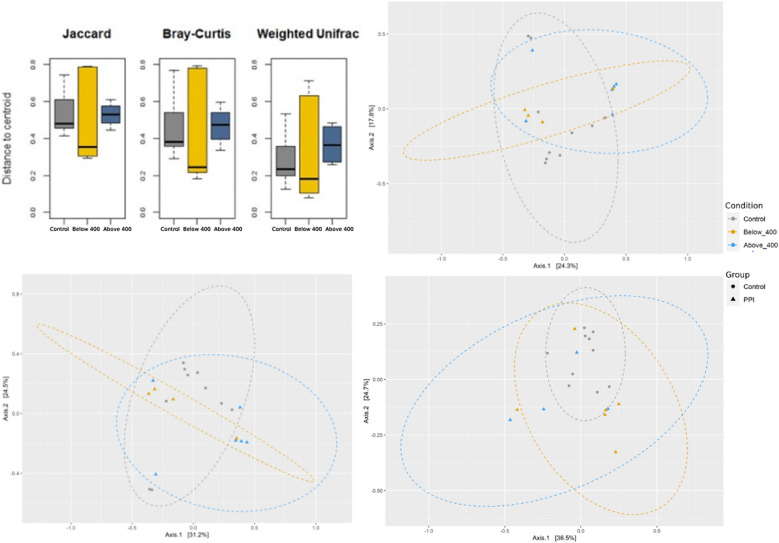
Beta diversity of the group exposed to proton pump inhibitors 1 month after treatment cessation, by treatment duration (below or above 400 days) and compared to the control group. Top left: dispersion of samples from centroid point for specific beta diversity metrics. Top right: PCoA plot of bray Curtis matrix; Bottom left: PCoA plot of Jaccard; Bottom right: PCoA plot of weighted unifrac

Beta-diversity analyses comparing the PPI groups with the controls were conducted for the baseline samples (Additional file 4: Fig. S4) and for 1 month after PPI treatment cessation. Figure 4A shows that the distance to centroid when clustering the data is bigger for infants treated less than 400 days with PPI than infants treated below 400 days, but interestingly, the group treated with less the 400 days of PPI are more like the controls (same direction of the axis—Fig. 4B–D). The weighted unifrac distance analysis adds to the analysis the phylogenetic composition of the data, which suggests that the below 400 days group seems more like the control group (yet with large variation).

### Specific species

**Fig. 5:**
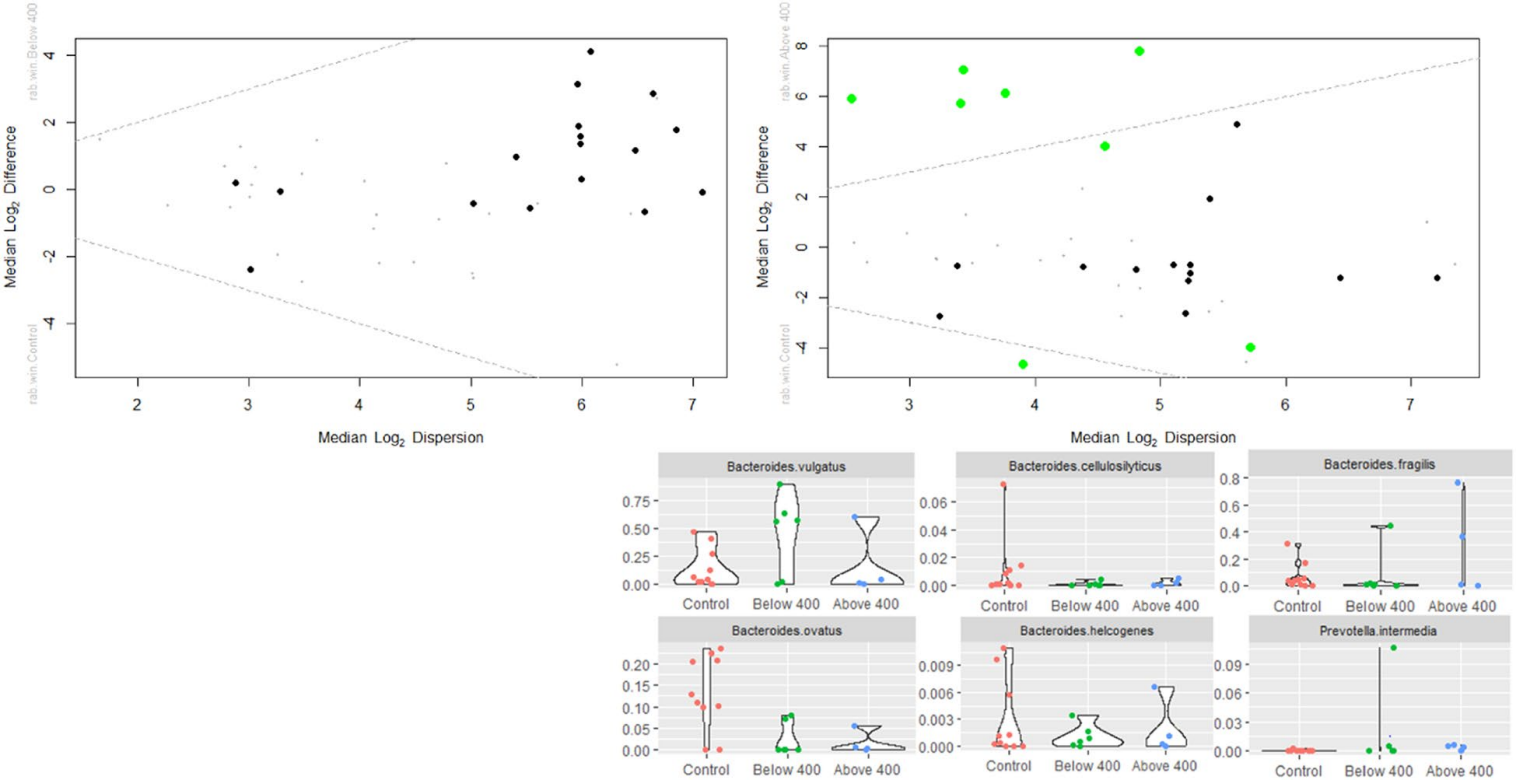
Differential abundance test comparing group exposed to proton pump inhibitors (PPI) 1 month after finalization of treatment, by treatment duration and compared to control group. Top left group below 400 days of PPI exposure versus control; top right group above 400 days of PPI versus control, Bottom right the 6 bacteria differentially expressed when comparing PPI and control group

The differential abundance test using ALDEx2 analysis compares pairs of conditions for the last timepoints for the groups below and above 400 days with the controls (Fig. 5). For the group above 400 days, six bacterial species was differentially expressed compared to the control group (*Bacteroides vulgatus, Bacteroides cellulosilyticus, Bacteroides fragilis, Bacteroides ovatus, Bacteroides helcogenes* and *Prevotella intermedia)*; while no differentially expressed bacteria were identified for the below 400 days group. The differential abundance tests comparing the baseline (total PPI group) with the controls are shown in Additional file 5: Fig. S5 indicating 4 bacteria were differentially expressed *(Clostridium bolteae* (or *Enterocloster bolteae*), *Bacteroides ovatus*, *Bacteroides cellulosilyticus* and *Bacteroides helcogenes)*. No differences were found when comparing group below and above 400 days at last time point (Additional file 6: Fig. S6). An overview of these species and their association with diseases and health conditions as described in the literature, is presented in the Additional file 11: Table S2.

### After treatment cessation

Additional file 7: Figures S7, Additional file 8: Fig. S8 visualize the different species by treatment duration (below or above 400 days) 1 month after treatment cessation, compared to the controls.

### Antibiotic use and antibiotic resistance profiling

No differences were observed when analyzing antibiotic consumption frequency. Assessment of antibiotic resistance profiling shows how many hits (more than 80% similarity) each sample had, compared to genes involved in one of the assessed pathways or resistance. At this early stage, it seems that the antibiotic did not cause any major persistent changes in the antibiotic resistance patterns between the three groups (short and long PPI duration, and ALADDIN controls—Additional file 9: Fig. S9).

## Discussion

This small yet unique cohort of young infants exposed to long duration of PPI use suggests that PPIs may alter the gut microbiome composition, with longer durations being potentially more harmful than shorter durations. Longer duration of PPIs was associated with considerable changes in evenness and unstable diversity compared to a shorter duration of use. We found no associations with the number of antibiotic treatment episodes among the PPI users. Although the microbiome composition of both PPI groups differed from those in the control infants, these findings should be interpreted with caution due to the slightly different sampling methodology and other confounders, including batch effects and storage conditions. Previous work has suggested that processing can exacerbate infant differences, exceeding biological effects of interest [56]. Also, the cohort size needs to be considered when interpreting the results since only ten infants were exposed to PPIs in this study, which especially limits interpretation.

The main strengths of the study include the homogenous group of newborn children, all with the same indication for PPI use (EA), which are all followed closely; and a control group with a similar lifestyle and socio-geographic background (all living in Sweden within a radius of 100 km). In addition, collection, processing and storage of all fecal samples were standardized, and state-of-the-art microbiome methods (Shotgun metagenomics) were used [56, 57]. Comparison of two different cohorts works under three assumptions: (1) infant microbiomes and trajectories are consistent; (2) there is no between study effect or between study effect will be negligible in the population; and (3) the PPI-related treatment signal is large enough that it will exceed interpersonal variation in this population. Although the gut microbiome is known to change dramatically during the first years of life [57–59], samples were collected at the same ages of the infants at regular intervals. External modifiers, such as antibiotic intake, were restricted by only including control children not exposed to antibiotics; and all infants were born vaginally [58]. The infants with EA did receive breastmilk from their own mothers as soon as they started feeding (7–10 days after surgery). Yet, duration of nursing with breastmilk has not been collected, nor timing and type of complementary feeding [57]. In addition, the noted differences between children with and without EA may be related to the anatomical differences (including a potential higher risk of gastroesophageal reflux [17, 26]) and other treatment characteristics besides the PPI use since the control children are clearly healthier. Unfortunately, the depth we applied for sequencing, does not allow further assessment of functionality. To have confidence at gene-level, we would need to sequence several times deeper. The method we used for DNA extraction is not appropriate for lysis of the fungi. Therefore, although we do get some species, the data are skewed, making it inappropriate for fungi analysis.

Direct evidence on the effect of PPIs on the microbiome in children remains limited [29], with two studies based on 16 s rRNA sequencing including (some) small children [54, 55]. One study followed 12 infants with confirmed gastro-esophageal reflux, exposed to oral PPIs for 8–44 weeks [54]. The authors concluded that PPI use had a minor impact on their fecal microbiome [54]. Yet, these children were older at the time of PPI initiation than the children with EA in our study (mean age of 5.2 months, range 0.5–10.2 months), and had a shorter duration of PPI exposure (mean 18 weeks compared to 57 weeks) [54]. Another study including 20 children (age 1–18 years, mean 5.8 years) exposed to PPIs for 4–8 weeks for various indications, did not find strong evidence for changes in their gut microbiome [55]. A study on older children (1–18 years, mean age 7 years) compared those exposed to PPIs during the last 48 h (N = 59,), and reported changes in the gastric, lung and oropharyngeal microbiome compared to children not using PPIs (N = 57) recently [60].

Our findings may add to the cumulative body of evidence warning against the wide-spread, and especially prolonged use of PPIs, especially if efficacy remains questionable. Although we still know insufficiently about the long-term effects, a precautionary approach may be warranted—limiting prescription to well-described indications for which efficacy has been established and restricting dose and duration if possible [61, 62]. Well-designed clinical trials seem warranted to assess the benefits and risk of PPIs after EA repair, in particular to establish a minimal duration of use.

## Conclusion

This pilot study suggests that prolonged PPI use may alter the infant gut microbiome composition, especially among those with the longest duration of use. A more cautious approach in PPI-prescribing in children seems justified, especially in case of prolonged use.

## Methods

This pilot study, conducted in Uppsala University Children’s Hospital in Sweden, enrolled all consecutive children operated for EA with a lower tracheoesophageal fistula (Gross type C [63]) between May 2016 and June 2018 who participated in the national follow-up program for one-year-old children with EA. For this study, only children born through vaginal delivery with fecal microbiome collection occurring at least three times (see below) were included. Children treated with antibiotics within 3 months before sampling were excluded.

Intravenous antibiotics (Cefotaxime) started as soon as the diagnosis of EA was confirmed. A primary anastomosis between the upper and the lower esophageal pouch was achieved in all children in the study group. Intravenous PPI treatment (Esomeprazole (Nexium^®^) 1 mg/kg/day) was initiated after surgery and switched to oral administration when the infant started feeding. This was continued for at least one year, according to the European and North American guidelines for treating the treatment of esophageal atresia [17]. Antibiotic administration ended after a routine contrast esophagography 7–10 days after the surgery, and the infants started with oral feeding.

### External control group

As a control group, healthy one-year old infants never treated with PPIs were selected from the prospective Assessment of Lifestyle and Allergic Diseases During Infancy (ALADDIN) birth cohort, which was described in detail previously [64, 65]. In this study, 330 children (from the Stockholm region in Sweden) were followed up from pregnancy to the age of 24 months to assess differences in lifestyle factors and sensitization taking into account anthroposophic lifestyle [64, 65]. Pregnant women were recruited between September 2004 and November 2007. An extensive data-collection scheme was applied, including multiple questionnaires and biological samples [64, 65]. For the present study, 12 children from the ALADDIN cohort were selected from families with a conventional, non-anthroposophic lifestyle who did not receive antibiotics at least 6 months before sampling and were born at term through vaginal delivery.

### Data collection

Information was collected on sex, gestational age at birth, mode of delivery, duration of follow-up and use of PPIs and antibiotics.

The Regional Ethics Committee of Uppsala University (2017/181) and the Research Ethical Committee at Huddinge University Hospital, Stockholm, Sweden (2010/1811-32) approved this study. Written informed consent was obtained from all caregivers of the included infants.

### Sample collection

For the children with EA, fecal samples from three timepoints were evaluated: during PPI treatment, two weeks after (at one-year follow-up), and 1 month after discontinuation. All samples were sent by mail by the caregivers and stored at − 80° C within 2 days after collection.

In the ALADDIN study, feces were collected from the children at seven timepoints and frozen within 20 min of collection and stored at − 20 °C until later transported in a frozen state to storage at − 70 °C [64, 65]. For the present study, only the 12-month sample was used.

All samples are preserved in DNA/RNA shield (Zymo Research—R1100-250), which keeps the bacterial constitution stable at room temperature for up to 30 days. All samples were shipped frozen to the Centre for Translational Microbiome Research on dry ice and stored at − 80 °C until processing.

### Preparation of samples and microbiota composition assessment

DNA was extracted from the 250 mg stool samples using a dual physical and chemical lysis protocol with the Quick-DNA Magbead Plus kit (D4082; Zymo Research, Irvine, CA, USA). This protocol is adapted primarily for bacteria and archaea; fungi are rarely captured. Previous to extraction, the samples went through 1 min of bead-beating at 1600 rpm (ZR Bashing Bead lysis matrix—S6012; Zymo Research, Irvine, CA, USA) followed by 30 min of lysozyme solution treatment at 37 °C (lysozyme recipe: 20 mM Tris–Cl, pH 8; 2 mM sodium EDTA [Tris–EDTA; Sigma-Aldrich, catalogue no. T9285]; lysozyme [Sigma-Aldrich, catalogue no. L6876-100G] to 100 mg/ml) and proteinase K at 55 °C for 30 min (20 mg/ml, part of Zymo extraction kit). The extraction was done using an automated high-throughput pipeline for human microbiome sampling, as previously described [66]. Only samples exceeding 10,000 reads were used.

A total of 50 ng of DNA was used for sequencing. The sequencing was performed using MGI whole-genome sequencing technology (MGI FS DNA library prep kit (1,000,013,455—MGI, Shenzhen, China) and sequencing kit (PE150 1,000,016,952; MGI)) in a DNBSEQ-T7 sequencer MGI as previously described.[67] All controls from the extraction phase and a negative PCR control were submitted to PCR and sequenced with the samples. Data were then processed for quality filtering, trimming, human reads removal and assignment of remaining microbial reads to taxonomic groups using the STAG-mwc pipeline (version v0.4.1) [68].

The data were analyzed using several R packages [69–75]. Normalization was performed using the centered log-ratio (CLR) method. The vegan R package was used to evaluate alpha-diversity (i.e. how many different species could be detected in a microbial ecosystem) by using the *Shannon diversity index,* which measures both the number of species (richness) and the inequality between species abundances (diversity) [72, 73]. A large value is given by the presence of many species with well-balanced abundances; lower values denote a poor diversity (for example, in the case of a single dominant species), while higher values are related to several species presenting similar abundance. The *Pielou's evenness (or equitability) index* is a pure diversity index and measures how evenly the microbes are distributed in a sample without considering the number of species. Values can range from zero to one: from high dominance of a single species to perfectly equal abundances across all species. The third measure of alpha-diversity, *phylogenetic diversity (PD),* is a phylogenetic metric (i.e., based on abundance and phylogenetic information); which weigh relatively rare, mid-abundant and abundant species [67]. The phylogenetic metrics are suitable when associated species have disparity in abundance and phylogeny.

Beta-diversity (analyzed using phyloseq and CoDaSeq packages) or diversity between the samples was assessed by means of an ordination plot and *principal component analyses (PCoA)* plots which are exploratory data visualization tools [69–71]. In addition, we tested whether the samples cluster beyond that expected by sampling variability using permutational multivariate analysis of variance (*PERMANOVA*), by partitioning the sums of squares for the within- and between-cluster components using the concept of centroids. Many permutations (n = 999) of the data (i.e., random shuffling) are used to generate the null distribution [76]. The *Weighted UniFrac metric* was used to incorporate phylogenic information by calculating the total branch lengths “unshared” between two samples divided by the total branch length [77, 78]. This approach often reveals interesting differences in the phylogenic relatedness between samples and sample types. *Differential abundance tests* (ALDEx2 part of CoDaSeq package) were used to identify specific taxa associated with clinical metadata variables of interest. Finally, *antibiotic resistance* profiles were assessed to see how many hits each sample had to genes involved in one of these resistance pathways (using armplusplus part of Stag-mwc pipeline and PCoA).

The children with EA were divided into two groups based on a shorter (< 400 days) or longer duration (≥ 400 days) of PPI use; and compared to the ALADDIN controls to assess confounding based on pre-existing microbiome variation. Potential effects by sex of the infant (data not shown), and antibiotic use were also evaluated.

## Supplementary Information

**Additional file 1:** **Figure S1.** Stacked barplot for Control group versus Proton Pump Inhibitor (PPI) group at baseline and species level

**Additional file 2:** **Figure S2.** Alpha diversity of Proton Pump Inhibitor (PPI) group at baseline. Top left: Observed metrics; Top right: Shannon diversity; Bottom left: Pielou Evenness; Bottom right: Phylogenetic Diversity. Samples are colored according to the treatment (Control or PPI).* p<0.05 compared to control group.

**Additional file 3:** **Figure S3.** Alpha diversity of Proton Pump Inhibitor (PPI) group at final timepoint. Top left: Observed metrics; Top right: Shannon diversity; Bottom left: Pielou Evenness; Bottom right: Phylogenetic Diversity. Samples are colored according to the treatment (Control or PPI).* p<0.05 compared to control group.

**Additional file 4:** **Figure S4.** Beta diversity of Proton Pump Inhibitor (PPI) group at baseline, compared to controls. Top left: dispersion of samples from centroid point for specific beta diversity metric. Top right: PCoA plot of Bray Curtis Matric; Bottom left: PCoA plot of Jaccard; Bottom right: PCoA plot of weighted unifrac.

**Additional file 5:** **Figure S5.** Differential abundance test for Proton Pump Inhibitor (PPI) group at baseline, indicating four bacterial species being differentially expressed when compared to controls, by the duration of treatment.

**Additional file 6:** **Figure S6.** Differential abundance test for Proton Pump Inhibitor (PPI) group comparing those below and above 400 days of exposure, showing no difference between both groups.

**Additional file 7:** **Figure S7. **Stacked barplot showing controls, and both groups of Proton Pump Inhibitors (PPI) below and above 400 days of exposure one month after treatment cessation (timepoint 3).

**Additional file 8:** **Figure S8.** Alpha diversity of baseline for control group, and both groups of Proton Pump Inhibitors (PPI) below and above 400 days of exposure, one month after treatment cessation (timepoint 3). Top left: observed metrics, Top right: Shannon diversity; Bottom left: Pielou Evenness; Bottom right: phylogenetic diversity. Samples are colored according to the exposure (and duration) to PPI.

**Additional file 9:** **Figure S9.** Antibiotic treatment and antibiotic resistance. No significant difference or clustering is observable based on the number of antibiotic interventions

**Additional file 10:** **Table S1.** Taxon name.

**Additional file 11:** **Table S2.** Comparing control and PPI children at baseline.

## Data Availability

The dataset(s), including the sequences supporting the conclusions of this article, is(are) available in the ENA repository, [PRJEB56662], and filtered reads matrix is available as Additional file 10: Table S1a. All methods were carried out in accordance with relevant guidelines and regulations**.**

## References

[1] Liu Y, Zhu X, Li R, Zhang J, Zhang F (2020). Proton pump inhibitor utilisation and potentially inappropriate prescribing analysis: insights from a single-centred retrospective study. BMJ Open.

[2] Pottegard A, Broe A, Hallas J, de Muckadell OB, Lassen AT, Lodrup AB (2016). Use of proton-pump inhibitors among adults: a Danish nationwide drug utilization study. Ther Adv Gastroenterol.

[3] Kim J, Blackett JW, Jodorkovsky D (2018). Strategies for effective discontinuation of proton pump inhibitors. Curr Gastroenterol Rep.

[4] Forgacs I, Loganayagam A (2008). Overprescribing proton pump inhibitors. BMJ.

[5] Boghossian TA, Rashid FJ, Thompson W, Welch V, Moayyedi P, Rojas-Fernandez C (2017). Deprescribing versus continuation of chronic proton pump inhibitor use in adults. Cochrane Database Syst Rev.

[6] Sears K, Elms S, Whitehead M, Tranmer JE, Edge DS, VanDenKerkhof EG (2019). A population-based study of prescribing trends in a potentially vulnerable paediatric population from 1999 to 2012. Int J Pharm Pract.

[7] Hudson B, Alderton A, Doocey C, Nicholson D, Toop L, Day AS (2012). Crying and spilling—time to stop the overmedicalisation of normal infant behaviour. N Z Med J.

[8] Putnam PE (2009). Stop the PPI express: they don't keep babies quiet!. J Pediatr.

[9] O'Reilly D, Conway R, O'Connor L, Fitzpatrick P (2020). Use of anti-reflux medications in infants under 1 year of age: a retrospective drug utilization study using national prescription reimbursement data. Eur J Pediatr.

[10] Zhou Y, Xu L, Wushouer H, Yu A, Xu Z, Chen C (2021). Acid suppression use among infants in one tertiary children’s hospital in China, 2015–2018: a retrospective observational study. Front Pediatr.

[11] Simon M, Levy EI, Vandenplas Y (2021). Safety considerations when managing gastro-esophageal reflux disease in infants. Expert Opin Drug Saf.

[12] Zoizner-Agar G, Rotsides JM, Shao Q, Rickert S, Ward R, Greifer M (2020). Proton pump inhibitor administration in neonates and infants. Lack of consensus—an ASPO survey. Int J Pediatr Otorhinolaryngol.

[13] Palčevski G, Skočibušić N, Vlahović-Palčevski V (2012). Unlicensed and off-label drug use in hospitalized children in Croatia: a cross-sectional survey. Eur J Clin Pharmacol.

[14] Ruíz-Antorán B, Piñeiro R, Avendaño C, Román E, Cilleruelo ML, Gutiérrez-Junquera C (2013). Drug utilization and off-label drug use in Spanish pediatric gastroenterology outpatients. J Pediatr Gastroenterol Nutr.

[15] Ellwood J, Draper-Rodi J, Carnes D (2020). Comparison of common interventions for the treatment of infantile colic: a systematic review of reviews and guidelines. BMJ Open.

[16] Yao DWJ, Ong C, Eales NM, Sultana R, Wong JJ, Lee JH (2021). Reassessing the use of proton pump inhibitors and histamine-2 antagonists in critically Ill children: a systematic review and meta-analysis. J Pediatr.

[17] Krishnan U, Mousa H, Dall'Oglio L, Homaira N, Rosen R, Faure C (2016). ESPGHAN-NASPGHAN guidelines for the evaluation and treatment of gastrointestinal and nutritional complications in children with esophageal atresia-tracheoesophageal fistula. J Pediatr Gastroenterol Nutr.

[18] Halfdanarson OO, Pottegard A, Bjornsson ES, Lund SH, Ogmundsdottir MH, Steingrimsson E (2018). Proton-pump inhibitors among adults: a nationwide drug-utilization study. Ther Adv Gastroenterol.

[19] Luo H, Fan Q, Xiao S, Chen K (2018). Changes in proton pump inhibitor prescribing trend over the past decade and pharmacists’ effect on prescribing practice at a tertiary hospital. BMC Health Serv Res.

[20] Zeng W, Finlayson AE, Shankar S, de Bruyn W, Godman B (2015). Prescribing efficiency of proton pump inhibitors in China: influence and future directions. BMC Health Serv Res.

[21] Barron JJ, Tan H, Spalding J, Bakst AW, Singer J (2007). Proton pump inhibitor utilization patterns in infants. J Pediatr Gastroenterol Nutr.

[22] De Bruyne P, Christiaens T, Vander Stichele R, Van Winckel M (2014). Changes in prescription patterns of acid-suppressant medications by Belgian pediatricians: analysis of the national database, [1997–2009]. J Pediatr Gastroenterol Nutr.

[23] Donoso F, Lilja HE (2017). Risk factors for anastomotic strictures after esophageal atresia repair: prophylactic proton pump inhibitors do not reduce the incidence of strictures. Eur J Pediatr Surg.

[24] The KP, Working PPIIN (2021). Assess don’t guess. Gastroenterology.

[25] Righini Grunder F, Petit LM, Ezri J, Jantchou P, Aspirot A, Laberge S (2019). Should proton pump inhibitors be systematically prescribed in patients with esophageal atresia after surgical repair?. J Pediatr Gastroenterol Nutr.

[26] Miyake H, Chen Y, Hock A, Seo S, Koike Y, Pierro A (2018). Are prophylactic anti-reflux medications effective after esophageal atresia repair? Systematic review and meta-analysis. Pediatr Surg Int.

[27] Kuhn BR, Young AJ, Justice AE, Chittoor G, Walton NA (2020). Infant acid suppression use is associated with the development of eosinophilic esophagitis. Dis Esophagus.

[28] Oshima T, Wu L, Li M, Fukui H, Watari J, Miwa H (2018). Magnitude and direction of the association between *Clostridium*
*difficile* infection and proton pump inhibitors in adults and pediatric patients: a systematic review and meta-analysis. J Gastroenterol.

[29] Levy EI, Hoang DM, Vandenplas Y (2020). The effects of proton pump inhibitors on the microbiome in young children. Acta Paediatr.

[30] Mitre E, Susi A, Kropp LE, Schwartz DJ, Gorman GH, Nylund CM (2018). Association between use of acid-suppressive medications and antibiotics during infancy and allergic diseases in early childhood. JAMA Pediatr.

[31] Vijay G, Mandal A, Sankar J, Kapil A, Lodha R, Kabra SK (2018). Ventilator associated pneumonia in pediatric intensive care unit: incidence, risk factors and etiological agents. Indian J Pediatr.

[32] Wang YH, Wintzell V, Ludvigsson JF, Svanstrom H, Pasternak B (2021). Association between proton pump inhibitor use and risk of asthma in children. JAMA Pediatr.

[33] Eusebi LH, Rabitti S, Artesiani ML, Gelli D, Montagnani M, Zagari RM (2017). Proton pump inhibitors: risks of long-term use. J Gastroenterol Hepatol.

[34] Xie Y, Bowe B, Yan Y, Xian H, Li T, Al-Aly Z (2019). Estimates of all cause mortality and cause specific mortality associated with proton pump inhibitors among US veterans: cohort study. BMJ.

[35] Sasaki T, Mori S, Kishi S, Fujiwara-Tani R, Ohmori H, Nishiguchi Y (2020). Effect of proton pump inhibitors on colorectal cancer. Int J Mol Sci.

[36] Segna D, Brusselaers N, Glaus D, Krupka N, Misselwitz B (2021). Association between proton-pump inhibitors and the risk of gastric cancer: a systematic review with meta-analysis. Ther Adv Gastroenterol.

[37] Devine RE, McCleary N, Sheikh A, Nwaru BI (2017). Acid-suppressive medications during pregnancy and risk of asthma and allergy in children: a systematic review and meta-analysis. J Allergy Clin Immunol.

[38] Breddels E, Simin J, Fornes R, Lilja HE, Engstrand L, Bruyndonckx R, et al. Population-based cohort study: Proton pump inhibitor use during pregnancy in Sweden and the risk of maternal and neonatal adverse events. BMC Med. 2022, in press. 10.1186/s12916-022-02673-x 10.1186/s12916-022-02673-x PMC9768950 36539798

[39] Li CM, Zhernakova A, Engstrand L, Wijmenga C, Brusselaers N (2020). Systematic review with meta-analysis: the risks of proton pump inhibitors during pregnancy. Aliment Pharmacol Ther.

[40] Malfertheiner P, Kandulski A, Venerito M (2017). Proton-pump inhibitors: understanding the complications and risks. Nat Rev Gastroenterol Hepatol.

[41] Dominguez-Bello MG, Godoy-Vitorino F, Knight R, Blaser MJ (2019). Role of the microbiome in human development. Gut.

[42] Turroni F, Milani C, Duranti S, Lugli GA, Bernasconi S, Margolles A (2020). The infant gut microbiome as a microbial organ influencing host well-being. Ital J Pediatr.

[43] Kapourchali FR, Cresci GAM (2020). Early-life gut microbiome-the importance of maternal and infant factors in its establishment. Nutr Clin Pract.

[44] Falony G, Joossens M, Vieira-Silva S, Wang J, Darzi Y, Faust K (2016). Population-level analysis of gut microbiome variation. Science.

[45] Le Bastard Q, Al-Ghalith GA, Gregoire M, Chapelet G, Javaudin F, Dailly E (2018). Systematic review: human gut dysbiosis induced by non-antibiotic prescription medications. Aliment Pharmacol Ther.

[46] Zhernakova A, Kurilshikov A, Bonder MJ, Tigchelaar EF, Schirmer M, Vatanen T (2016). Population-based metagenomics analysis reveals markers for gut microbiome composition and diversity. Science.

[47] Bruno G, Zaccari P, Rocco G, Scalese G, Panetta C, Porowska B (2019). Proton pump inhibitors and dysbiosis: current knowledge and aspects to be clarified. World J Gastroenterol.

[48] Bender JM, Li F, Purswani H, Capretz T, Cerini C, Zabih S (2019). Early exposure to antibiotics in the neonatal intensive care unit alters the taxonomic and functional infant gut microbiome. J Matern Fetal Neonatal Med.

[49] Kim CS, Grady N, Derrick M, Yu Y, Oliphant K, Lu J (2021). Effect of antibiotic use within first 48 hours of life on the preterm infant microbiome: a randomized clinical trial. JAMA Pediatr.

[50] Wong WSW, Sabu P, Deopujari V, Levy S, Shah AA, Clemency N (2020). Prenatal and peripartum exposure to antibiotics and cesarean section delivery are associated with differences in diversity and composition of the infant meconium microbiome. Microorganisms..

[51] Bokulich NA, Chung J, Battaglia T, Henderson N, Jay M, Li H (2016). Antibiotics, birth mode, and diet shape microbiome maturation during early life. Sci Transl Med.

[52] Mueller NT, Whyatt R, Hoepner L, Oberfield S, Dominguez-Bello MG, Widen EM (2015). Prenatal exposure to antibiotics, cesarean section and risk of childhood obesity. Int J Obes.

[53] Schulfer A, Blaser MJ (2015). Risks of antibiotic exposures early in life on the developing microbiome. PLoS Pathog.

[54] Castellani C, Singer G, Kashofer K, Huber-Zeyringer A, Flucher C, Kaiser M (2017). The influence of proton pump inhibitors on the fecal microbiome of infants with gastroesophageal reflux—a prospective longitudinal interventional study. Front Cell Infect Microbiol.

[55] Simakachorn L, Tanpowpong P, Chanprasertyothin S, Thongpradit S, Treepongkaruna S (2021). Gut microbiota characteristics in children after the use of proton pump inhibitors. Turk J Gastroenterol.

[56] Salter SJ, Cox MJ, Turek EM, Calus ST, Cookson WO, Moffatt MF (2014). Reagent and laboratory contamination can critically impact sequence-based microbiome analyses. BMC Biol.

[57] Backhed F, Roswall J, Peng Y, Feng Q, Jia H, Kovatcheva-Datchary P (2015). Dynamics and stabilization of the human gut microbiome during the first year of life. Cell Host Microbe.

[58] Dominguez-Bello MG, Blaser MJ, Ley RE, Knight R (2011). Development of the human gastrointestinal microbiota and insights from high-throughput sequencing. Gastroenterology.

[59] Koenig JE, Spor A, Scalfone N, Fricker AD, Stombaugh J, Knight R (2011). Succession of microbial consortia in the developing infant gut microbiome. Proc Natl Acad Sci U S A.

[60] Rosen R, Hu L, Amirault J, Khatwa U, Ward DV, Onderdonk A (2015). 16S community profiling identifies proton pump inhibitor related differences in gastric, lung, and oropharyngeal microflora. J Pediatr.

[61] Vergouwe FW, Gottrand M, Wijnhoven BP, Ijsselstijn H, Piessen G, Bruno MJ (2018). Four cancer cases after esophageal atresia repair: time to start screening the upper gastrointestinal tract. World J Gastroenterol.

[62] Vergouwe FWT, IJsselstijn H, Biermann K, Erler NS, Wijnen RMH, Bruno MJ (2018). High prevalence of Barrett's esophagus and esophageal squamous cell carcinoma after repair of esophageal atresia. Clin Gastroenterol Hepatol.

[63] Gross R (1953). The surgery of infancy and childhood: its principles and techniques.

[64] Hesla HM, Stenius F, Jäderlund L, Nelson R, Engstrand L, Alm J (2014). Impact of lifestyle on the gut microbiota of healthy infants and their mothers—the ALADDIN birth cohort. FEMS Microbiol Ecol.

[65] Stenius F, Swartz J, Lilja G, Borres M, Bottai M, Pershagen G (2011). Lifestyle factors and sensitization in children—the ALADDIN birth cohort. Allergy.

[66] Hugerth L, Seifert M, Pennhag A, Du J, Hamsten M, Schuppe-Koistinen I (2018). A comprehensive automated pipeline for human microbiome sampling, 16S rRNA gene sequencing and bioinformatics processing. bioRxiv..

[67] Hugerth LW, Pereira M, Zha Y, Seifert M, Kaldhusdal V, Boulund F (2020). Assessment of in vitro and in silico protocols for sequence-based characterization of the human vaginal microbiome. mSphere..

[68] Boulund F, Debelius J, Olsson L. ctmrbio/stag-mwc: StaG v0.4.1 (Version v0.4.1). Zenodo. 2021.

[69] Badri M, Kurtz Z, Muller C, Bonneau R (2018). bioRxiv..

[70] Gloor GB, Reid G (2016). Compositional analysis: a valid approach to analyze microbiome high-throughput sequencing data. Can J Microbiol.

[71] Gloor GB, Wu JR, Pawlowsky-Glahn V, Egozcue JJ (2016). It's all relative: analyzing microbiome data as compositions. Ann Epidemiol.

[72] McMurdie PJ, Holmes S (2014). Waste not, want not: why rarefying microbiome data is inadmissible. PLoS Comput Biol.

[73] Oksanen J, Blanchet G, Friendly M, Kindt R, Legendre P, McGlinn D, et al. vegan: Community Ecology Package. R package version 2.5–7. 2020.

[74] Kembel SW, Cowan PD, Helmus MR, Cornwell WK, Morlon H, Ackerly DD (2010). Picante: R tools for integrating phylogenies and ecology. Bioinformatics.

[75] Palarea Albaladejo J, Martín-Fernández J (2015). zCompositions—R package for multivariate imputation of left-censored data under a compositional approach. Chemom Intell Lab Syst.

[76] Faith DP (1992). Conservation evaluation and phylogenetic diversity. Biol Cons.

[77] Lozupone C, Knight R (2005). UniFrac: a new phylogenetic method for comparing microbial communities. Appl Environ Microbiol.

[78] Lozupone CA, Hamady M, Kelley ST, Knight R (2007). Quantitative and qualitative beta diversity measures lead to different insights into factors that structure microbial communities. Appl Environ Microbiol.

