# Supplementary figures and images for: Effect of proton pump inhibitors in infants with esophageal atresia on the gut microbiome: a pilot cohort
Source: Gut Pathog. 2022 Dec 16;14:47. doi: 10.1186/s13099-022-00518-9 (PMC9758939; doi:10.1186/s13099-022-00518-9)

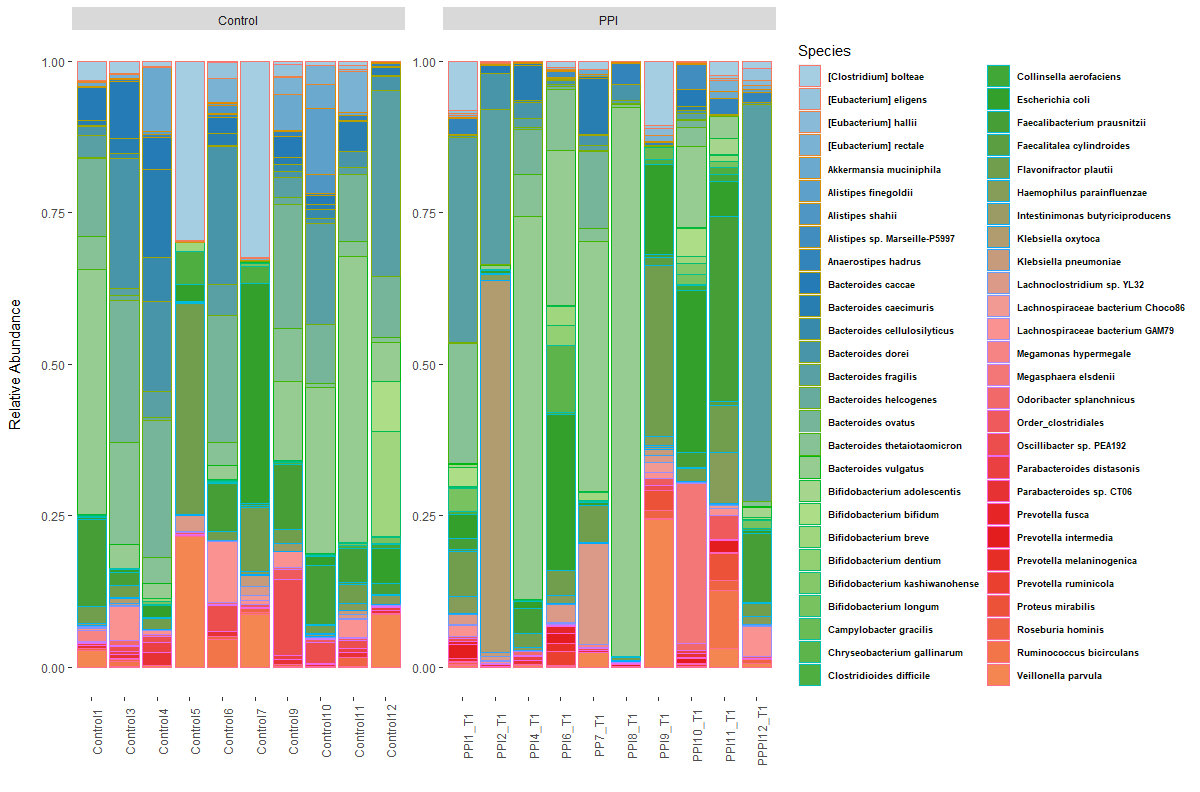

Supplement: Supplementary file 1 — Additional file 1: Figure S1. Stacked barplot for Control group versus Proton Pump Inhibitor (PPI) group at baseline and species level [file 13099_2022_518_MOESM1_ESM.png]

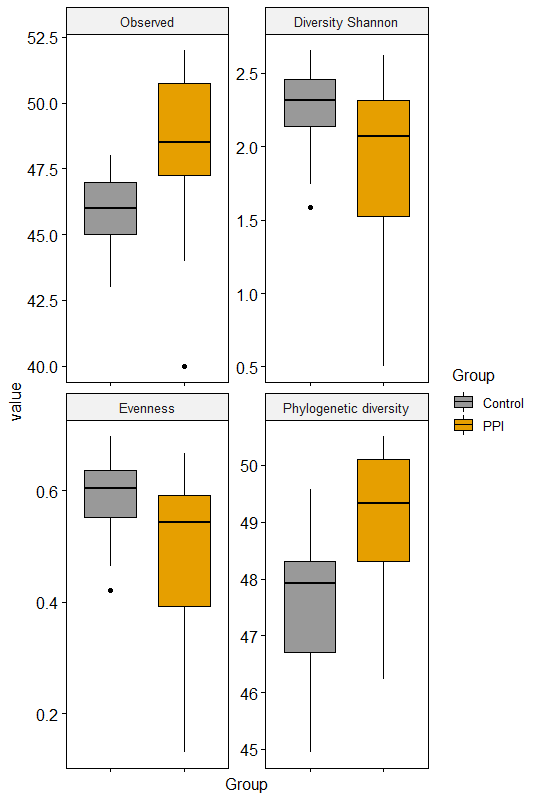

Supplement: Supplementary file 2 — Additional file 2: Figure S2. Alpha diversity of Proton Pump Inhibitor (PPI) group at baseline. Top left: Observed metrics; Top right: Shannon diversity; Bottom left: Pielou Evenness; Bottom right: Phylogenetic Diversity. Samples are colored according to the treatment (Control or PPI).* p<0.05 compared to control group. [file 13099_2022_518_MOESM2_ESM.png]

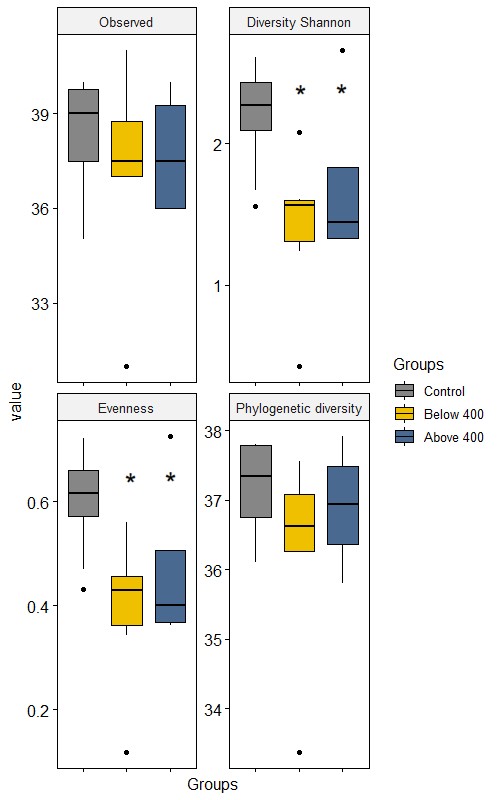

Supplement: Supplementary file 3 — Additional file 3: Figure S3. Alpha diversity of Proton Pump Inhibitor (PPI) group at final timepoint. Top left: Observed metrics; Top right: Shannon diversity; Bottom left: Pielou Evenness; Bottom right: Phylogenetic Diversity. Samples are colored according to the treatment (Control or PPI).* p<0.05 compared to control group. [file 13099_2022_518_MOESM3_ESM.jpg]

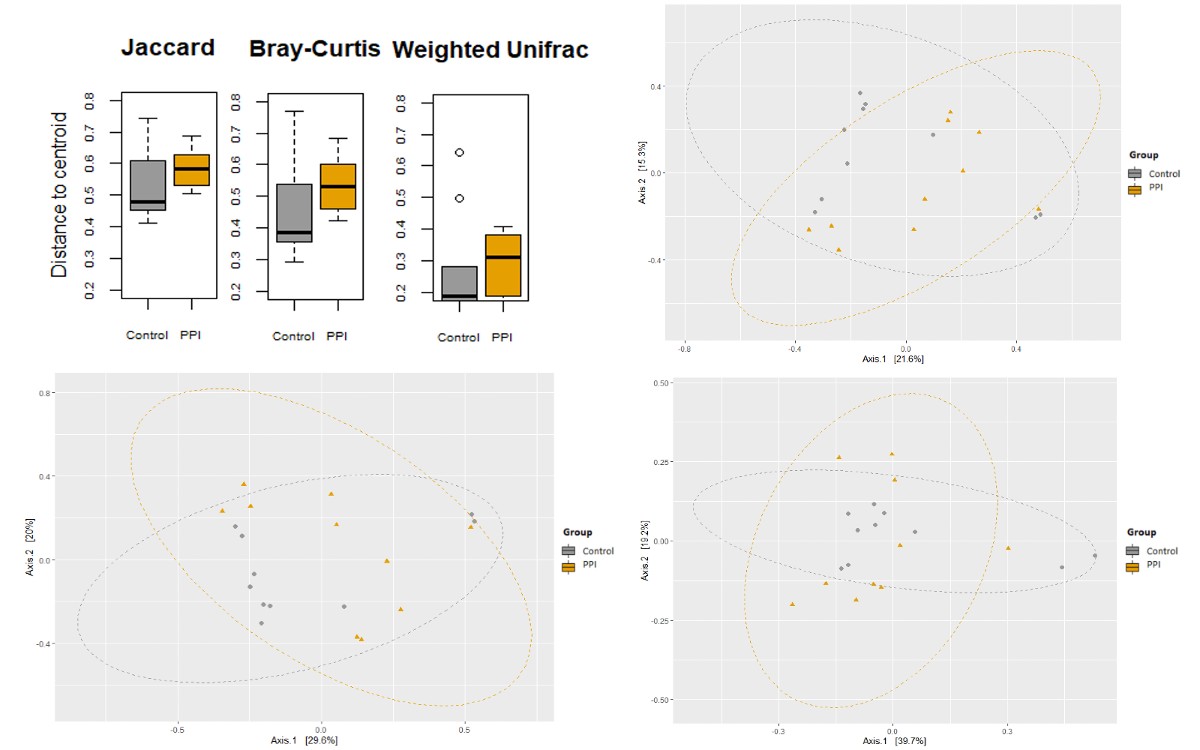

Supplement: Supplementary file 4 — Additional file 4: Figure S4. Beta diversity of Proton Pump Inhibitor (PPI) group at baseline, compared to controls. Top left: dispersion of samples from centroid point for specific beta diversity metric. Top right: PCoA plot of Bray Curtis Matric; Bottom left: PCoA plot of Jaccard; Bottom right: PCoA plot of weighted unifrac. [file 13099_2022_518_MOESM4_ESM.jpg]

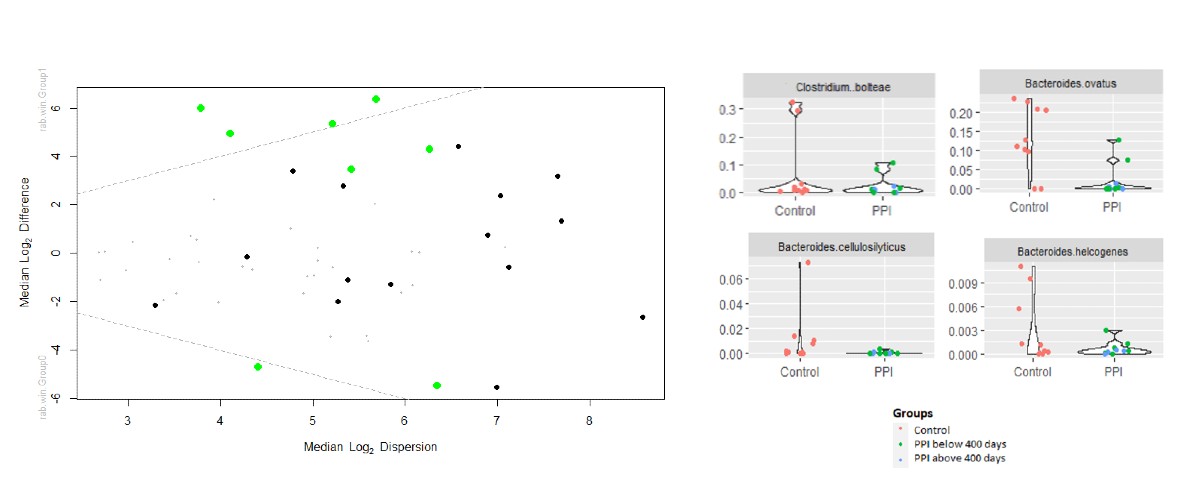

Supplement: Supplementary file 5 — Additional file 5: Figure S5. Differential abundance test for Proton Pump Inhibitor (PPI) group at baseline, indicating four bacterial species being differentially expressed when compared to controls, by the duration of treatment. [file 13099_2022_518_MOESM5_ESM.jpg]

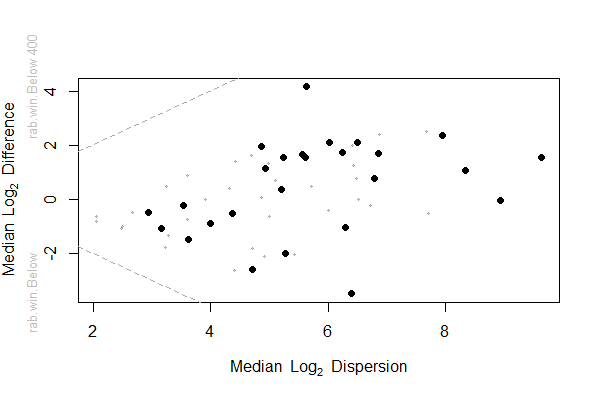

Supplement: Supplementary file 6 — Additional file 6: Figure S6. Differential abundance test for Proton Pump Inhibitor (PPI) group comparing those below and above 400 days of exposure, showing no difference between both groups. [file 13099_2022_518_MOESM6_ESM.tiff]

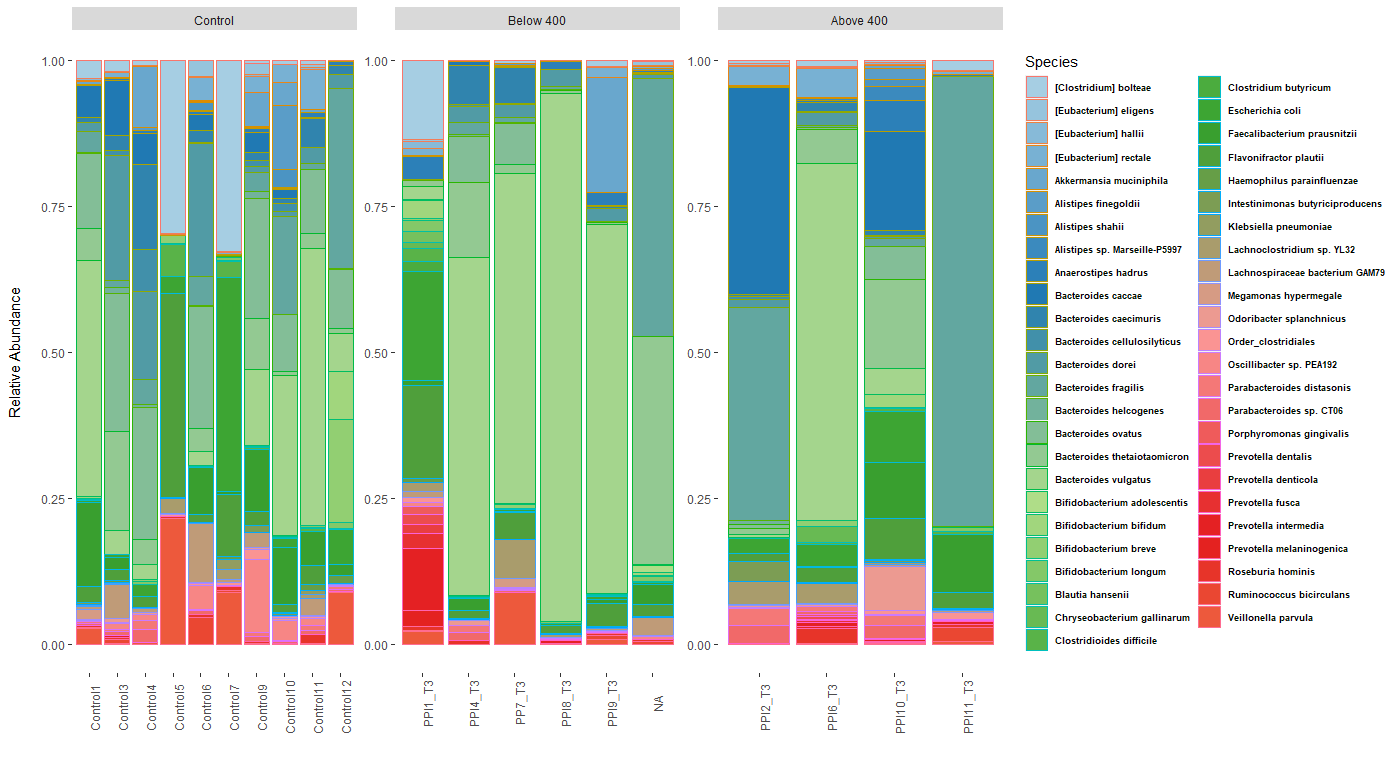

Supplement: Supplementary file 7 — Additional file 7: Figure S7. Stacked barplot showing controls, and both groups of Proton Pump Inhibitors (PPI) below and above 400 days of exposure one month after treatment cessation (timepoint 3). [file 13099_2022_518_MOESM7_ESM.png]

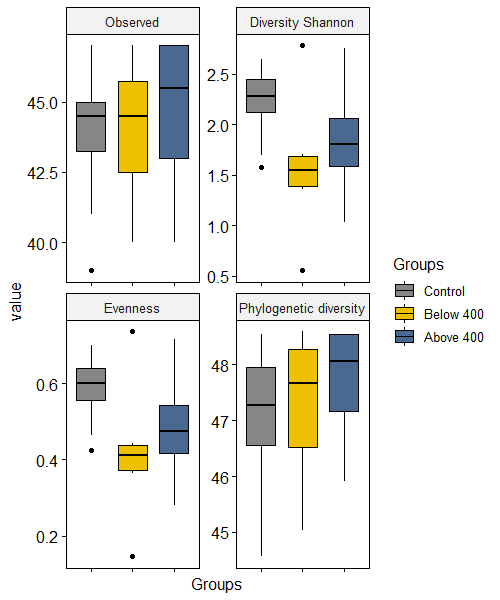

Supplement: Supplementary file 8 — Additional file 8: Figure S8. Alpha diversity of baseline for control group, and both groups of Proton Pump Inhibitors (PPI) below and above 400 days of exposure, one month after treatment cessation (timepoint 3). Top left: observed metrics, Top right: Shannon diversity; Bottom left: Pielou Evenness; Bottom right: phylogenetic diversity. Samples are colored according to the exposure (and duration) to PPI. [file 13099_2022_518_MOESM8_ESM.png]

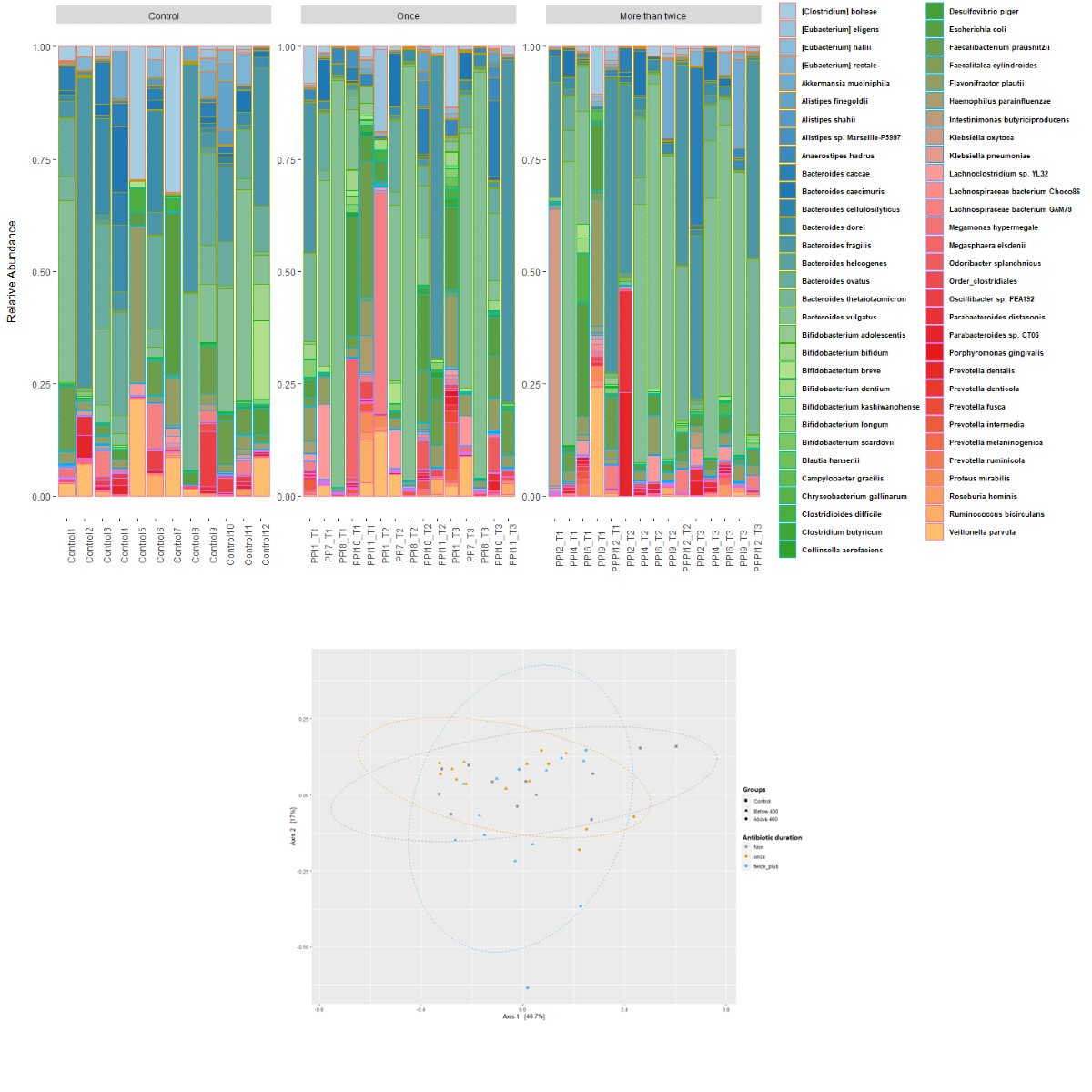

Supplement: Supplementary file 9 — Additional file 9: Figure S9. Antibiotic treatment and antibiotic resistance. No significant difference or clustering is observable based on the number of antibiotic interventions [file 13099_2022_518_MOESM9_ESM.jpg]
