# Supplementary material for: Effect of proton pump inhibitors in infants with esophageal atresia on the gut microbiome: a pilot cohort
Source: Gut Pathog. 2022 Dec 16;14:47. doi: 10.1186/s13099-022-00518-9 (PMC9758939; doi:10.1186/s13099-022-00518-9)
Supplement: Supplementary file 11 — Additional file 11: Table S2. Comparing control and PPI children at baseline. [file 13099_2022_518_MOESM11_ESM.docx]

| **Comparing control and PPI children at baseline** | | | | | | | | |
| --- | --- | --- | --- | --- | --- | --- | --- | --- |
| **Original name** | **Other names** | **Gram +/-** | **Form** | **(An)aerobic** | **Isolated from humans** | **Other diseases association** | **Reference** |  |
| *Clostridium bolteae (*↓*)* | ***Enterocloster bolteae*** | + | Rod shaped | Obligate anaerobic | 2003(1) | Autism (↑) | Song et al 2004(1) |  |
|  |  |  |  |  |  |  | Kandaeel et al 2020(2) |  |
|  |  |  |  |  |  | Systemic sclerosis (↑) | Plichta et al 2021(3) |  |
|  |  |  |  |  |  | Food allergy (↑) | Kourosh et al 2018(4) |  |
|  |  |  |  |  |  | Spondylarthritis (↑) | Wang et al 2022(5) |  |
|  |  |  |  |  |  | Rheumatoid arthritis (↑) | Kim et al 2021(6) |  |
| *Bacteroides ovatus (↓)* | NA | - | Bacillus | Obligate anaerobic | 1933(7) | Decreases gut inflammation (↑) | Ihekweazu et al 2021(8) |  |
|  |  |  |  |  |  | IgA production (↑) | Yang et al 2020(9) |  |
|  |  |  |  |  |  | IBD (↑) | Saitoh et al 2002(10) |  |
|  |  |  |  |  |  | Functional constipation (↑) | De Meij et al 2016(11) |  |
|  |  |  |  |  |  | Coeliac disease (↓) | Sanchez et al 2010(12) |  |
|  |  |  |  |  |  | Recurrent Clostridioides difficile infection (↓) | Mullish et al 2019(13) |  |
|  |  |  |  |  |  |  | Amrane et al 2019(14) |  |
|  |  |  |  |  |  | Shorter PFS in melanoma (↑) | Peters at al 2019(15) |  |
|  |  |  |  |  |  | Modulatory role on cancer therapy outcome (↑) | Heshiki et al 2020(16) |  |
|  |  |  |  |  |  | SARS-CoV-2 viral load (↓) | Zuo et al 2020(17) |  |
|  |  |  |  |  |  | Nonalcoholic fatty liver disease (↑) | Jiao et al 2021(18) |  |
| *Bacteroides cellulosilyticus (↓)* | NA | - | Bacillus | Obligate anaerobic | 2007(19) | Collagenous colitis (↑) | Gustafsson et al 2012(20) |  |
|  |  |  |  |  |  | Severe SARS-CoV-2 (↑) | Xu et al 2022(21) |  |
| *Bacteroides helcogenes (↓)* | NA | - | Bacillus | Obligate anaerobic | 1983 in pigs(22) | Mental disorders (↓) | McGuinness et al 2022(23) |  |
|  |  |  |  |  | 2006 in humans(24) | Vaginal delivery (↑) | Hanachi et al 2022(25) |  |
|  |  |  |  |  |  |  |  |  |

| **Comparing control and PPI children at the end of PPI treatment (only above 400 days if PPI treatment)** | | | | | | | | | |  |  |
| --- | --- | --- | --- | --- | --- | --- | --- | --- | --- | --- | --- |
| **Original name** | **Other names** | **Gram +/-** | **Form** | **(An)aerobic** | **Isolated from humans** | **Other diseases association** | | **Reference** |  |  |  |
| *Bacteroides vulgatus (↓)* | ***Phocaeicola vulgatus*** | - | Bacillus | Obligate anaerobic | 1933(7) | ME/CFS + IBD (↑) | | Nagy-Szakal et al 2017(26) |  |  |  |
|  |  |  |  |  |  | Crohn´s disease (↑) | | Mangin et al 2004(27) |  |  |  |
|  |  |  |  |  |  |  |  | Ruseler-van Embden and van Lieshout 1987(28) |  |  |  |
|  |  |  |  |  |  | Viral Diarehea (↓) | | Ma et al 2011(29) |  |  |  |
|  |  |  |  |  |  | Type 2 diabetes (↓) | | Wu et al 2010(30) |  |  |  |
|  |  |  |  |  |  | Low risk of irAEs during cancer treatment (↑) | | Usyk et al 2021(31) |  |  |  |
|  |  |  |  |  |  | UC treatment - blockage of proteases (-) | | Mills et al 2022(32) |  |  |  |
|  |  |  |  |  |  | CRC (↓) | | Wang et al 2012(33) |  |  |  |
| *Bacteroides cellulosilyticus (↓)* | NA | - | Bacillus | Obligate anaerobic | 2007(19) | Collagenous colitis (↑) | | Gustafsson et al 2012(20) |  |  |  |
|  |  |  |  |  |  | Severe SARS-CoV-2 (↑) | | Xu et al 2022(21) |  |  |  |
| *Bacteroides fragilis (↑)* | ***Bacillus fragilis*** | - | Bacillus | Anaerobic | 1898(34) | CRC (↑) - ETBF | | Viljoen et al 2015(35) |  |  |  |
|  |  |  |  |  |  |  |  | Sears et al 2014(36) |  |  |  |
|  |  |  |  |  |  |  |  | Haghi et al 2019(37) |  |  |  |
|  |  |  |  |  |  |  |  | Kordahi et al 2021(38) |  |  |  |
|  |  |  |  |  |  |  |  | Khodaverdi et al 2021(39) |  |  |  |
|  |  |  |  |  |  | Autoimmune disease (↑) - ETBF | | Stewart et al 2018(40) |  |  |  |
|  |  |  |  |  |  | Diarrhea (↑) - ETBF | | Pathela et al 2005(41) |  |  |  |
|  |  |  |  |  |  |  |  | Sears et al 2008(36) |  |  |  |
|  |  |  |  |  |  | Immune response (-) | | Sjogren et al 2009(42) |  |  |  |
|  |  |  |  |  |  | IBD (↑) - ETBF | | Zitomersky et al 2013(43) |  |  |  |
|  |  |  |  |  |  |  |  | Chu et al 2016(44) |  |  |  |
|  |  |  |  |  |  | Asthma (↑) | | Vael et al 2008(45) |  |  |  |
| *Bacteroides ovatus (↓)* | NA | - | Bacillus | Obligate anaerobic | 1933(7) | Decreases gut inflammation (↑) | | Ihekweazu et al 2021(8) |  |  |  |
|  |  |  |  |  |  | IgA production (↑) | | Yang et al 2014(46) |  |  |  |
|  |  |  |  |  |  | IBD (↑) | | Saitoh et al 2002(10) |  |  |  |
|  |  |  |  |  |  | Functional constipation (↑) | | De Meij et al 2016(11) |  |  |  |
|  |  |  |  |  |  | Coeliac disease (↓) | | Sanchez et al 2010(12) |  |  |  |
|  |  |  |  |  |  | Recurrent Clostridioides difficile infection (↓) | | Mullish et al 2019(13) |  |  |  |
|  |  |  |  |  |  |  |  | Amrane et al 2019(14) |  |  |  |
|  |  |  |  |  |  | Shorter PFS in melanoma (↑) | | Peters at al 2019(15) |  |  |  |
|  |  |  |  |  |  | Modulatory role on cancer therapy outcome (↑) | | Heshiki et al 2020(16) |  |  |  |
|  |  |  |  |  |  | SARS-CoV-2 viral load (↓) | | Zuo et al 2020(17) |  |  |  |
|  |  |  |  |  |  | Nonalcoholic fatty liver disease (↑) | | Jiao et al 2021(18) |  |  |  |
| *Bacteroides helcogenes (↓)* | NA | - | Bacillus | Obligate anaerobic | 1983 in pigs(22) | Mental disorders (↓) | | McGuinness et al 2022(23) |  |  |  |
|  |  |  |  |  | 2006 in humans(24) | Vaginal delivery (↑) | | Hanachi et al 2022(25) |  |  |  |
| *Prevotella intermedia (↑)* | ***Bacteroides melaninogenicus subsp. intermedius*** | - | Rod shaped | Anaerobic | 1970(47) | Severe periodontitis (↑) | | Torrungruang et al 2015(48) |  |  |  |
|  |  |  |  |  |  |  |  | Yang et al 2014(46) |  |  |  |
|  | ***Bacteroides intermedius*** |  |  |  |  | Gout (↑) | | Liu et al 2018(49) |  |  |  |
|  |  |  |  |  |  | Familiar Subclinical Periodontal Disease (-) | | Fukui et al 1999(50) |  |  |  |
|  |  |  |  |  |  |  |  | |  | |  |

**↑ increased levels**

**↓ decreased levels**

**"-" Important to the specific health condition**

**IBD Irritable bowel disease**

**PFS progression-free survival**

**ME/CFS + IBD Myalgic encephalomyelitis/chronic fatigue syndrome**

**irAEs immune-related adverse events**

**UC ulcerative colitis**

**CRC colon rectal cancer**

**ETBF enterotoxigenic Bacteroides fragilis**

**References**

1. Song Y, Liu C, Finegold SM. Real-time PCR quantitation of clostridia in feces of autistic children. Appl Environ Microbiol. 2004 Nov;70(11):6459-65. PubMed PMID: 15528506. Pubmed Central PMCID: PMC525120. Epub 2004/11/06. eng.

2. Kandeel WA, Meguid NA, Bjorklund G, Eid EM, Farid M, Mohamed SK, et al. Impact of Clostridium Bacteria in Children with Autism Spectrum Disorder and Their Anthropometric Measurements. Journal of molecular neuroscience : MN. 2020 Jun;70(6):897-907. PubMed PMID: 32130666. Epub 2020/03/05.

3. Plichta DR, Somani J, Pichaud M, Wallace ZS, Fernandes AD, Perugino CA, et al. Congruent microbiome signatures in fibrosis-prone autoimmune diseases: IgG4-related disease and systemic sclerosis. Genome medicine. 2021 Feb 28;13(1):35. PubMed PMID: 33648559. Pubmed Central PMCID: PMC7919092. Epub 2021/03/03. eng.

4. Kourosh A, Luna RA, Balderas M, Nance C, Anagnostou A, Devaraj S, et al. Fecal microbiome signatures are different in food-allergic children compared to siblings and healthy children. Pediatric allergy and immunology : official publication of the European Society of Pediatric Allergy and Immunology. 2018 Aug;29(5):545-54. PubMed PMID: 29624747. Epub 2018/04/07. eng.

5. Wang L, Wang Y, Zhang P, Song C, Pan F, Li G, et al. Gut microbiota changes in patients with spondyloarthritis: A systematic review. Seminars in arthritis and rheumatism. 2022 Feb;52:151925. PubMed PMID: 34844732. Epub 2021/12/01. eng.

6. Kim JW, Jeong Y, Park SJ, Jin H, Lee J, Ju JH, et al. Influence of proton pump inhibitor or rebamipide use on gut microbiota of rheumatoid arthritis patients. Rheumatology (Oxford, England). 2021 Feb 1;60(2):708-16. PubMed PMID: 32789440. Epub 2020/08/14.

7. Eggerth AH, Gagnon BH. The Bacteroides of Human Feces. J Bacteriol. 1933 Apr;25(4):389-413. PubMed PMID: 16559622. Pubmed Central PMCID: PMC533498. Epub 1933/04/01.

8. Ihekweazu FD, Engevik MA, Ruan W, Shi Z, Fultz R, Engevik KA, et al. Bacteroides ovatus Promotes IL-22 Production and Reduces Trinitrobenzene Sulfonic Acid-Driven Colonic Inflammation. The American journal of pathology. 2021 Apr;191(4):704-19. PubMed PMID: 33516788. Pubmed Central PMCID: PMC8027925. Epub 2021/02/01. eng.

9. Yang C, Mogno I, Contijoch EJ, Borgerding JN, Aggarwala V, Li Z, et al. Fecal IgA Levels Are Determined by Strain-Level Differences in Bacteroides ovatus and Are Modifiable by Gut Microbiota Manipulation. Cell host & microbe. 2020 Mar 11;27(3):467-75.e6. PubMed PMID: 32075742. Pubmed Central PMCID: PMC7213796. Epub 2020/02/23. eng.

10. Saitoh S, Noda S, Aiba Y, Takagi A, Sakamoto M, Benno Y, et al. Bacteroides ovatus as the predominant commensal intestinal microbe causing a systemic antibody response in inflammatory bowel disease. Clinical and diagnostic laboratory immunology. 2002 Jan;9(1):54-9. PubMed PMID: 11777829. Pubmed Central PMCID: PMC119885. Epub 2002/01/05. eng.

11. de Meij TG, de Groot EF, Eck A, Budding AE, Kneepkens CM, Benninga MA, et al. Characterization of Microbiota in Children with Chronic Functional Constipation. PloS one. 2016;11(10):e0164731. PubMed PMID: 27760208. Pubmed Central PMCID: PMC5070844 technology (patent ‘Microbial population analysis’, WO2008/125365) and are co-founders of a spin-off company developing this technique into a clinical diagnostic product. This patent is only related to the microbiota profiling technique IS-pro. This does not alter our adherence to all PLOS ONE policies on sharing data and materials. Epub 2016/10/21. eng.

12. Sánchez E, Donat E, Ribes-Koninckx C, Calabuig M, Sanz Y. Intestinal Bacteroides species associated with coeliac disease. Journal of clinical pathology. 2010 Dec;63(12):1105-11. PubMed PMID: 20972239. Epub 2010/10/26. eng.

13. Mullish BH, McDonald JAK, Pechlivanis A, Allegretti JR, Kao D, Barker GF, et al. Microbial bile salt hydrolases mediate the efficacy of faecal microbiota transplant in the treatment of recurrent Clostridioides difficile infection. Gut. 2019 Oct;68(10):1791-800. PubMed PMID: 30816855. Pubmed Central PMCID: PMC6839797. Epub 2019/03/01. eng.

14. Amrane S, Hocquart M, Afouda P, Kuete E, Pham TP, Dione N, et al. Metagenomic and culturomic analysis of gut microbiota dysbiosis during Clostridium difficile infection. Scientific reports. 2019 Sep 5;9(1):12807. PubMed PMID: 31488869. Pubmed Central PMCID: PMC6728329. Epub 2019/09/07. eng.

15. Peters BA, Wilson M, Moran U, Pavlick A, Izsak A, Wechter T, et al. Relating the gut metagenome and metatranscriptome to immunotherapy responses in melanoma patients. Genome medicine. 2019 Oct 9;11(1):61. PubMed PMID: 31597568. Pubmed Central PMCID: PMC6785875. Epub 2019/10/11. eng.

16. Heshiki Y, Vazquez-Uribe R, Li J, Ni Y, Quainoo S, Imamovic L, et al. Predictable modulation of cancer treatment outcomes by the gut microbiota. Microbiome. 2020 Mar 5;8(1):28. PubMed PMID: 32138779. Pubmed Central PMCID: PMC7059390. Epub 2020/03/07. eng.

17. Zuo T, Zhang F, Lui GCY, Yeoh YK, Li AYL, Zhan H, et al. Alterations in Gut Microbiota of Patients With COVID-19 During Time of Hospitalization. Gastroenterology. 2020 Sep;159(3):944-55.e8. PubMed PMID: 32442562. Pubmed Central PMCID: PMC7237927. Epub 2020/05/23. eng.

18. Jiao N, Loomba R, Yang ZH, Wu D, Fang S, Bettencourt R, et al. Alterations in bile acid metabolizing gut microbiota and specific bile acid genes as a precision medicine to subclassify NAFLD. Physiological genomics. 2021 Aug 1;53(8):336-48. PubMed PMID: 34151600. Pubmed Central PMCID: PMC8424563. Epub 2021/06/22. eng.

19. Robert C, Chassard C, Lawson PA, Bernalier-Donadille A. Bacteroides cellulosilyticus sp. nov., a cellulolytic bacterium from the human gut microbial community. International journal of systematic and evolutionary microbiology. 2007 Jul;57(Pt 7):1516-20. PubMed PMID: 17625186. Epub 2007/07/13. eng.

20. Gustafsson RJ, Ohlsson B, Benoni C, Jeppsson B, Olsson C. Mucosa-associated bacteria in two middle-aged women diagnosed with collagenous colitis. World journal of gastroenterology. 2012 Apr 14;18(14):1628-34. PubMed PMID: 22529692. Pubmed Central PMCID: PMC3325529. Epub 2012/04/25. eng.

21. Xu X, Zhang W, Guo M, Xiao C, Fu Z, Yu S, et al. Integrated analysis of gut microbiome and host immune responses in COVID-19. Frontiers of medicine. 2022 Apr;16(2):263-75. PubMed PMID: 35258762. Pubmed Central PMCID: PMC8902486. Epub 2022/03/09. eng.

22. Benno Y, Watabe J, Mitsuoka T. Bacteroides pyogenes sp. nov., Bacteroides suis sp. nov., and Bacteroides helcogenes sp. nov., New Species from Abscesses and Feces of Pigs. Systematic and applied microbiology. 1983;4(3):396-407. PubMed PMID: 23194738. Epub 1983/01/01. eng.

23. McGuinness AJ, Davis JA, Dawson SL, Loughman A, Collier F, O'Hely M, et al. A systematic review of gut microbiota composition in observational studies of major depressive disorder, bipolar disorder and schizophrenia. Molecular psychiatry. 2022 Apr;27(4):1920-35. PubMed PMID: 35194166. Pubmed Central PMCID: PMC9126816 probiotic-based biotherapeutics. AL is a named inventor on a patent relating to Prevotella. FNJ has written two books for commercial publication. The remaining authors declare competing interests. Epub 2022/02/24. eng.

24. Bakir MA, Kitahara M, Sakamoto M, Matsumoto M, Benno Y. Bacteroides intestinalis sp. nov., isolated from human faeces. International journal of systematic and evolutionary microbiology. 2006 Jan;56(Pt 1):151-4. PubMed PMID: 16403880. Epub 2006/01/13. eng.

25. Hanachi M, Maghrebi O, Bichiou H, Trabelsi F, Bouyahia NM, Zhioua F, et al. Longitudinal and Comparative Analysis of Gut Microbiota of Tunisian Newborns According to Delivery Mode. Frontiers in microbiology. 2022;13:780568. PubMed PMID: 35547149. Pubmed Central PMCID: PMC9083410. Epub 2022/05/14. eng.

26. Nagy-Szakal D, Williams BL, Mishra N, Che X, Lee B, Bateman L, et al. Fecal metagenomic profiles in subgroups of patients with myalgic encephalomyelitis/chronic fatigue syndrome. Microbiome. 2017 Apr 26;5(1):44. PubMed PMID: 28441964. Pubmed Central PMCID: PMC5405467. Epub 2017/04/27. eng.

27. Mangin I, Bonnet R, Seksik P, Rigottier-Gois L, Sutren M, Bouhnik Y, et al. Molecular inventory of faecal microflora in patients with Crohn's disease. FEMS microbiology ecology. 2004 Oct 1;50(1):25-36. PubMed PMID: 19712374. Epub 2004/10/01. eng.

28. Ruseler-van Embden JG, van Lieshout LM. Increased faecal glycosidases in patients with Crohn's disease. Digestion. 1987;37(1):43-50. PubMed PMID: 3609504. Epub 1987/01/01. eng.

29. Ma C, Wu X, Nawaz M, Li J, Yu P, Moore JE, et al. Molecular characterization of fecal microbiota in patients with viral diarrhea. Current microbiology. 2011 Sep;63(3):259-66. PubMed PMID: 21739252. Epub 2011/07/09. eng.

30. Wu X, Ma C, Han L, Nawaz M, Gao F, Zhang X, et al. Molecular characterisation of the faecal microbiota in patients with type II diabetes. Current microbiology. 2010 Jul;61(1):69-78. PubMed PMID: 20087741. Epub 2010/01/21. eng.

31. Usyk M, Pandey A, Hayes RB, Moran U, Pavlick A, Osman I, et al. Bacteroides vulgatus and Bacteroides dorei predict immune-related adverse events in immune checkpoint blockade treatment of metastatic melanoma. Genome medicine. 2021 Oct 13;13(1):160. PubMed PMID: 34641962. Pubmed Central PMCID: PMC8513370. Epub 2021/10/14. eng.

32. Mills RH, Dulai PS, Vázquez-Baeza Y, Sauceda C, Daniel N, Gerner RR, et al. Multi-omics analyses of the ulcerative colitis gut microbiome link Bacteroides vulgatus proteases with disease severity. Nature microbiology. 2022 Feb;7(2):262-76. PubMed PMID: 35087228. Pubmed Central PMCID: PMC8852248. Epub 2022/01/29. eng.

33. Wang T, Cai G, Qiu Y, Fei N, Zhang M, Pang X, et al. Structural segregation of gut microbiota between colorectal cancer patients and healthy volunteers. The ISME journal. 2012 Feb;6(2):320-9. PubMed PMID: 21850056. Pubmed Central PMCID: PMC3260502. Epub 2011/08/19. eng.

34. Veillon A, Zuber M. Recherches sur quelques microbes strictement anaérobies et leur rôle en pathologie. . Arch Med Exp. 1898;10:517–45.

35. Viljoen KS, Dakshinamurthy A, Goldberg P, Blackburn JM. Quantitative profiling of colorectal cancer-associated bacteria reveals associations between fusobacterium spp., enterotoxigenic Bacteroides fragilis (ETBF) and clinicopathological features of colorectal cancer. PloS one. 2015;10(3):e0119462. PubMed PMID: 25751261. Pubmed Central PMCID: PMC4353626. Epub 2015/03/10. eng.

36. Sears CL, Islam S, Saha A, Arjumand M, Alam NH, Faruque AS, et al. Association of enterotoxigenic Bacteroides fragilis infection with inflammatory diarrhea. Clinical infectious diseases : an official publication of the Infectious Diseases Society of America. 2008 Sep 15;47(6):797-803. PubMed PMID: 18680416. Pubmed Central PMCID: PMC3045827. Epub 2008/08/06. eng.

37. Haghi F, Goli E, Mirzaei B, Zeighami H. The association between fecal enterotoxigenic B. fragilis with colorectal cancer. BMC cancer. 2019 Sep 5;19(1):879. PubMed PMID: 31488085. Pubmed Central PMCID: PMC6727388. Epub 2019/09/07. eng.

38. Kordahi MC, Stanaway IB, Avril M, Chac D, Blanc MP, Ross B, et al. Genomic and functional characterization of a mucosal symbiont involved in early-stage colorectal cancer. Cell host & microbe. 2021 Oct 13;29(10):1589-98.e6. PubMed PMID: 34536346. Pubmed Central PMCID: PMC8979638. Epub 2021/09/19. eng.

39. Khodaverdi N, Zeighami H, Jalilvand A, Haghi F, Hesami N. High frequency of enterotoxigenic Bacteroides fragilis and Enterococcus faecalis in the paraffin-embedded tissues of Iranian colorectal cancer patients. BMC cancer. 2021 Dec 22;21(1):1353. PubMed PMID: 34937552. Pubmed Central PMCID: PMC8693489. Epub 2021/12/24. eng.

40. Stewart L, J DME, Blakely G, Patrick S. Antigenic mimicry of ubiquitin by the gut bacterium Bacteroides fragilis: a potential link with autoimmune disease. Clinical and experimental immunology. 2018 Nov;194(2):153-65. PubMed PMID: 30076785. Pubmed Central PMCID: PMC6194340. Epub 2018/08/05. eng.

41. Pathela P, Hasan KZ, Roy E, Alam K, Huq F, Siddique AK, et al. Enterotoxigenic Bacteroides fragilis-associated diarrhea in children 0-2 years of age in rural Bangladesh. The Journal of infectious diseases. 2005 Apr 15;191(8):1245-52. PubMed PMID: 15776370. Epub 2005/03/19. eng.

42. Sjögren YM, Tomicic S, Lundberg A, Böttcher MF, Björkstén B, Sverremark-Ekström E, et al. Influence of early gut microbiota on the maturation of childhood mucosal and systemic immune responses. Clinical and experimental allergy : journal of the British Society for Allergy and Clinical Immunology. 2009 Dec;39(12):1842-51. PubMed PMID: 19735274. Epub 2009/09/09. eng.

43. Zitomersky NL, Atkinson BJ, Franklin SW, Mitchell PD, Snapper SB, Comstock LE, et al. Characterization of adherent bacteroidales from intestinal biopsies of children and young adults with inflammatory bowel disease. PloS one. 2013;8(6):e63686. PubMed PMID: 23776434. Pubmed Central PMCID: PMC3679120. Epub 2013/06/19. eng.

44. Chu H, Khosravi A, Kusumawardhani IP, Kwon AH, Vasconcelos AC, Cunha LD, et al. Gene-microbiota interactions contribute to the pathogenesis of inflammatory bowel disease. Science. 2016 May 27;352(6289):1116-20. PubMed PMID: 27230380. Pubmed Central PMCID: PMC4996125. Epub 2016/05/28. eng.

45. Vael C, Nelen V, Verhulst SL, Goossens H, Desager KN. Early intestinal Bacteroides fragilis colonisation and development of asthma. BMC pulmonary medicine. 2008 Sep 26;8:19. PubMed PMID: 18822123. Pubmed Central PMCID: PMC2562360. Epub 2008/09/30. eng.

46. Yang NY, Zhang Q, Li JL, Yang SH, Shi Q. Progression of periodontal inflammation in adolescents is associated with increased number of Porphyromonas gingivalis, Prevotella intermedia, Tannerella forsythensis, and Fusobacterium nucleatum. International journal of paediatric dentistry. 2014 May;24(3):226-33. PubMed PMID: 24025042. Epub 2013/09/13. eng.

47. Holdeman LV, Moore WEC. Eubacterium. In Outline of Clinical Methods in Anaerobic Bacteriology. 2nd edition ed. Cato EP, Cummings CS, Holdeman LV, Johnsson JL, Moore WEC, Smibert RM, et al., editors: Virginia Polytechnic Institute Anaerobe Laborator

1970.

48. Torrungruang K, Jitpakdeebordin S, Charatkulangkun O, Gleebbua Y. Porphyromonas gingivalis, Aggregatibacter actinomycetemcomitans, and Treponema denticola / Prevotella intermedia Co-Infection Are Associated with Severe Periodontitis in a Thai Population. PloS one. 2015;10(8):e0136646. PubMed PMID: 26313005. Pubmed Central PMCID: PMC4552424. Epub 2015/08/28. eng.

49. Liu J, Cui L, Yan X, Zhao X, Cheng J, Zhou L, et al. Analysis of Oral Microbiota Revealed High Abundance of Prevotella Intermedia in Gout Patients. Cellular physiology and biochemistry : international journal of experimental cellular physiology, biochemistry, and pharmacology. 2018;49(5):1804-12. PubMed PMID: 30231244. Epub 2018/09/20. eng.

50. Fukui K, Kato N, Kato H, Watanabe K, Tatematsu N. Incidence of Prevotella intermedia and Prevotella nigrescens carriage among family members with subclinical periodontal disease. Journal of clinical microbiology. 1999 Oct;37(10):3141-5. PubMed PMID: 10488167. Pubmed Central PMCID: PMC85513. Epub 1999/09/17. eng.
